# AI-based design of a nuclear reactor core

**DOI:** 10.1038/s41598-021-98037-1

**Published:** 2021-10-04

**Authors:** Vladimir Sobes, Briana Hiscox, Emilian Popov, Rick Archibald, Cory Hauck, Ben Betzler, Kurt Terrani

**Affiliations:** 1 University of Tennessee, Knoxville, USA; 2 Oak Ridge National Laboratory, Oak Ridge, USA; 3 Ultra Safe Nuclear Corporation, Oak Ridge, USA

**Keywords:** Nuclear energy, Energy science and technology, Engineering, Power stations, Mathematics and computing, Applied mathematics, Computational science, Scientific data

## Abstract

The authors developed an artificial intelligence (AI)-based algorithm for the design and optimization of a nuclear reactor core based on a flexible geometry and demonstrated a 3× improvement in the selected performance metric: temperature peaking factor. The rapid development of advanced, and specifically, additive manufacturing (3-D printing) and its introduction into advanced nuclear core design through the Transformational Challenge Reactor program have presented the opportunity to explore the arbitrary geometry design of nuclear-heated structures. The primary challenge is that the arbitrary geometry design space is vast and requires the computational evaluation of many candidate designs, and the multiphysics simulation of nuclear systems is very time-intensive. Therefore, the authors developed a machine learning-based multiphysics emulator and evaluated thousands of candidate geometries on Summit, Oak Ridge National Laboratory’s leadership class supercomputer. The results presented in this work demonstrate temperature distribution smoothing in a nuclear reactor core through the manipulation of the geometry, which is traditionally achieved in light water reactors through variable assembly loading in the axial direction and fuel shuffling during refueling in the radial direction. The conclusions discuss the future implications for nuclear systems design with arbitrary geometry and the potential for AI-based autonomous design algorithms.

## Introduction

The rapid development of advanced manufacturing and its application to advanced reactor design in the Transformational Challenge Reactor (TCR) program^1^ have presented the opportunity to explore the potential revolutionary benefits of the arbitrary geometry design of nuclear systems. Nuclear engineering design is no longer bound to the simple geometries manufacturable by traditional methods, slabs, cylinders, and spheres (e.g., fuel plates, fuel pellets, fuel pebbles)^2,3^. However, the increased freedom of designing an arbitrary geometry system comes at the cost of an increased complexity in design optimization. Dimensionality quickly makes the design problem overwhelming for engineers. To address this issue, the authors implemented an artificial intelligence (AI)-based optimization algorithm and established a challenge problem to demonstrate the application.

**Figure 1:**
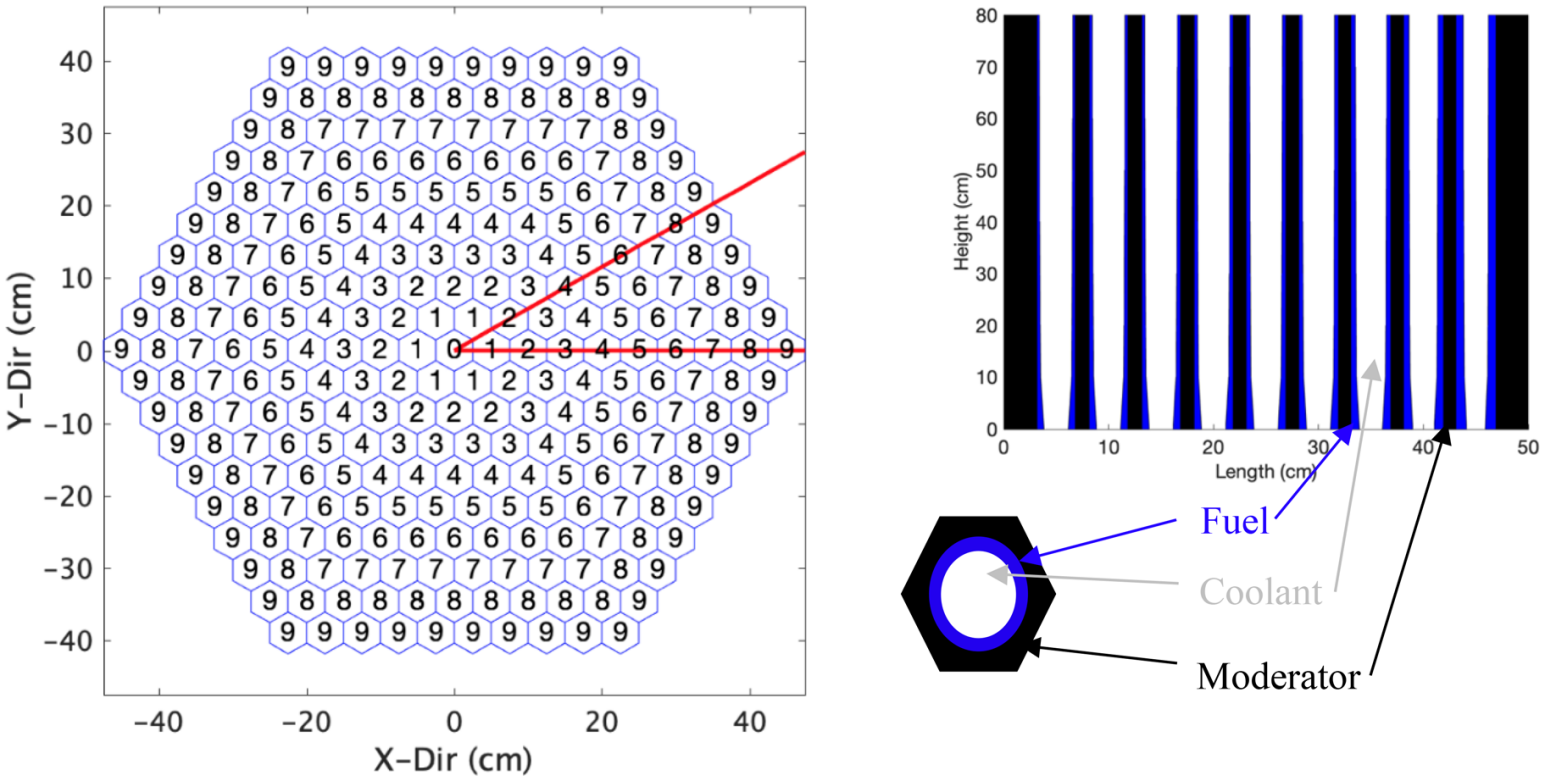
The top view of the core is shown on the left with the nine radial assembly rings labeled. Only the symmetric 1/12 segment inside the red lines is modeled. A horizontal slice of one assembly and an axial cross section of the optimized core configuration is shown on the right. Created using MATLAB R2020b, www.mathworks.com.

The basis of the challenge problem is to determine the optimal geometric shape in the axial dimension of the cooling channels of a simplified reactor design’s full-core model. The reactor core used in this AI design optimization is based on a simplification of the design of the actual TCR core. The core is a right cylinder that is 1 m in diameter and 80 cm tall. Nine concentric rings of hexagonal assemblies surround a central hexagonal assembly. The fuel compact of traditional TRISO particles is an annular design with a 2.5 cm outer diameter, and sits in the middle of each 5 cm wide (flat to flat) hexagonal assembly. There are cooling channels inside the annular fuel, and an yttrium-hydride the moderator surrounds the outside of the fuel annulus. The center assembly in the core is pure moderator, uncooled and unfueled. The helium coolant flows from the bottom to the top in all the fueled assemblies. Figure 1 presents a core schematic.

The design space encompassed the axial profile of the coolant channel in each of the nine assembly radial rings. That is, all the assemblies in each ring had the same axial coolant channel profile, but the coolant channel profile was different for each radial assembly ring. The geometry optimization was parametrized by a set of coolant channel radii. Each of the nine assembly radial rings had an independent set of nine coolant channel radii which spanned the 80 cm height of the core in 10 cm vertical segments. The radium of the coolant channel was a piece-wise linear function connecting these nine radii for each assembly. The maximum coolant channel radius was constrained to be greater than 1 mm and 2.4 cm (1 mm less than the fuel outer radius).

The objective for this core design was to minimize the temperature peaking factor across each 10 cm vertical segment (eight making the full axial height) of each assembly while maintaining a critical core configuration. The engineering justification for this objective function was to minimize the mechanical stresses due to temperature gradients in the components, although no thermomechanical analyses were performed in this initial study. Specifically, the objective function is defined as:

1 $$F\left( \Phi \right) = \frac{1}{{10}}\sum\limits_{{i = 1}}^{{10}} {\left\{ {\max T\left( {x,y,z,\Phi } \right) - \min T\left( {x,y,z,\Phi } \right):8\left( {i - 1} \right)~ cm \le z \le 8i ~ cm,(x,y,z) \in Fuel} \right\}} ,$$

where $T\left(x,y,z,\Phi \right)$ is the temperature at the location $\left(x,y,z\right)$ and design parameters $\Phi$.

The challenge problem inherently requires multiphysics modeling between neutron transport and thermofluidics. The problem is further convoluted because the cooling channel radius (axially variable) simultaneously controls everything about the heat transfer process and the amount of fuel at each axial core level because the fuel annulus outer diameter is fixed. The predictive simulation of candidate configurations requires computationally intensive modeling. The authors used a Monte Carlo-based code for neutron transport coupled to a computational fluid dynamics (CFD) code for the thermofluidics. With a large potential design space to explore, it was impractical to evaluate all of the candidate designs with the full fidelity physics. Therefore, the authors developed a machine learning (ML)-based multiphysics emulator that was designed to run on Summit, Oak Ridge National Laboratory’s (ORNL’s) GPU-based high-performance computing (HPC) system^4^. By training the ML-based emulator, the authors achieved errors as low as a few percent, which allowed them to quickly and reliably sample thousands of candidate designs on Summit. Only the most promising candidate designs were tested with the full-fidelity physics simulations. The emulator was updated, and convergence on the optimal design was achieved in only a few iterations.

The “Background” section reviews of some of the previous attempts at AI-based nuclear reactor design and optimization that did not leverage the advantages of arbitrary geometry. The section also discusses designing ML-based emulators for computationally intensive full-fidelity modeling software. The “Methods” section describes the overall workflow of the authors’ approach and presents the details of the full-fidelity modeling, the design of the emulator, and the optimization approach used on Summit. The “Results” section presents and discusses the optimal design solution to the challenge problem. Lastly, the “Discussion” section concludes with the authors’ outlook on the future of nuclear systems design in arbitrary geometry and discusses how the AI-based nuclear systems design approach can supplement the toolbox of nuclear industry designers.

## Background

Today, reactor cores are built from industrial materials that represent regular (i.e., usually cylindrical or plate) component geometries. The reactor cores have a periodic structure; for example, one component (i.e., core fuel element) is repeated multiple times to create the entire core. This approach results in geometrically similar fuel elements with a regular shape^5^. The fuel volumetric (per unit volume of core) content is spatially uniform. This limitation causes complications and results in fuel usage with different enrichment to efficiently design the entire core, minimizing power and thermal gradients. Similar observations can be made regarding the hydraulic design. The core is cooled to remove heat and generate power. The periodic repeatable fuel structure imminently produces the same regular coolant channel configuration (e.g., all coolant channels are the same and will produce the same level of cooling under the same conditions). The background of this research assumes an additively manufactured core in which the fuel and cooling channel geometries have practically unlimited spatial degrees of freedom. This is the primary difference explored in this work, and it might bring unforeseen performance that could be hidden from designers but easily discoverable for an unbiased AI algorithm.

Previous attempts at AI-based design and optimization of nuclear reactor cores are found in other works; e.g. References^6–13^. One common theme among these earlier works is that the optimization problem is posed as a combinatorial problem with fixed geometry rather than as an optimization over continuously variable geometry parametrization. One example is the fuel shuffling during the reloading of a boiling water reactor core. Genetic algorithms have been the predominant choice of AI algorithms for the combinatorial optimization problem. When more continuous variable geometries were studied, more structured optimization approaches were used^14^. A recent work considered the nuclear systems design by using genetic algorithms created from scratch rather than an optimization of an existing configuration^15^. However, arbitrary geometry could only be considered through a voxel representation. Lastly, the authors had published on the framework for an arbitrary geometry optimization of nuclear systems and gave some demonstrations on simplified challenge problems^2^. This work is the application of that framework to a nuclear reactor full core.

The mathematical formulations and their solutions for the underlying multiphysics phenomena that occur in the reactor core are well-known. For this work, the focus in on solutions of the Boltzmann transport equation, which will be the driving term to Poisson’s equation coupled with the Navier–Stokes equations. The solution to this set of coupled equations in nuclear engineering is tied up in several complex computer codes and is computationally intensive. Therefore, an efficient multiparameter optimization search using high-fidelity physics models is prohibitively expensive. In this work, the authors explored the automatic construction of physics-informed ML methods by using emulators with validation from very sparse sampling of the predictive high-fidelity physics simulations. The physics-informed emulators are based on a steady-state reduced-order model through Gaussian kernel convolution that allows for a fast evaluation on a single GPU^16^, which is suitable for ideal scalability in the search over the vast design space.

Surrogate modeling is a well-used method in science and engineering^17^. The fundamental approach is to approximate quantities of interest from complex systems by using cost-effective and accurate surrogates that otherwise could only be measured or simulated at very high cost, if at all. Within the context of design optimization, building cost-effective and accurate surrogates enables the exploration of complicated design space, accelerating the process of finding the best designs for given loss functions. The surrogate modeling method that the authors developed in this work falls under the category of multi-fidelity surrogate modeling. The authors merged the information generated by the dense sampling of reduced-order modeling with sparse full physics simulation via Gaussian processes (GPs)^18^. This work uniquely developed reduced-order models of the neutronics and thermofluidic physics that were designed for a fast evaluation on a single GPU. Thus, it is possible to evaluate millions of designs by using GPU-based HPC systems, such as Summit^4^.

## Methods

### Optimization work flow: inner and outer loops

**Figure 2:**
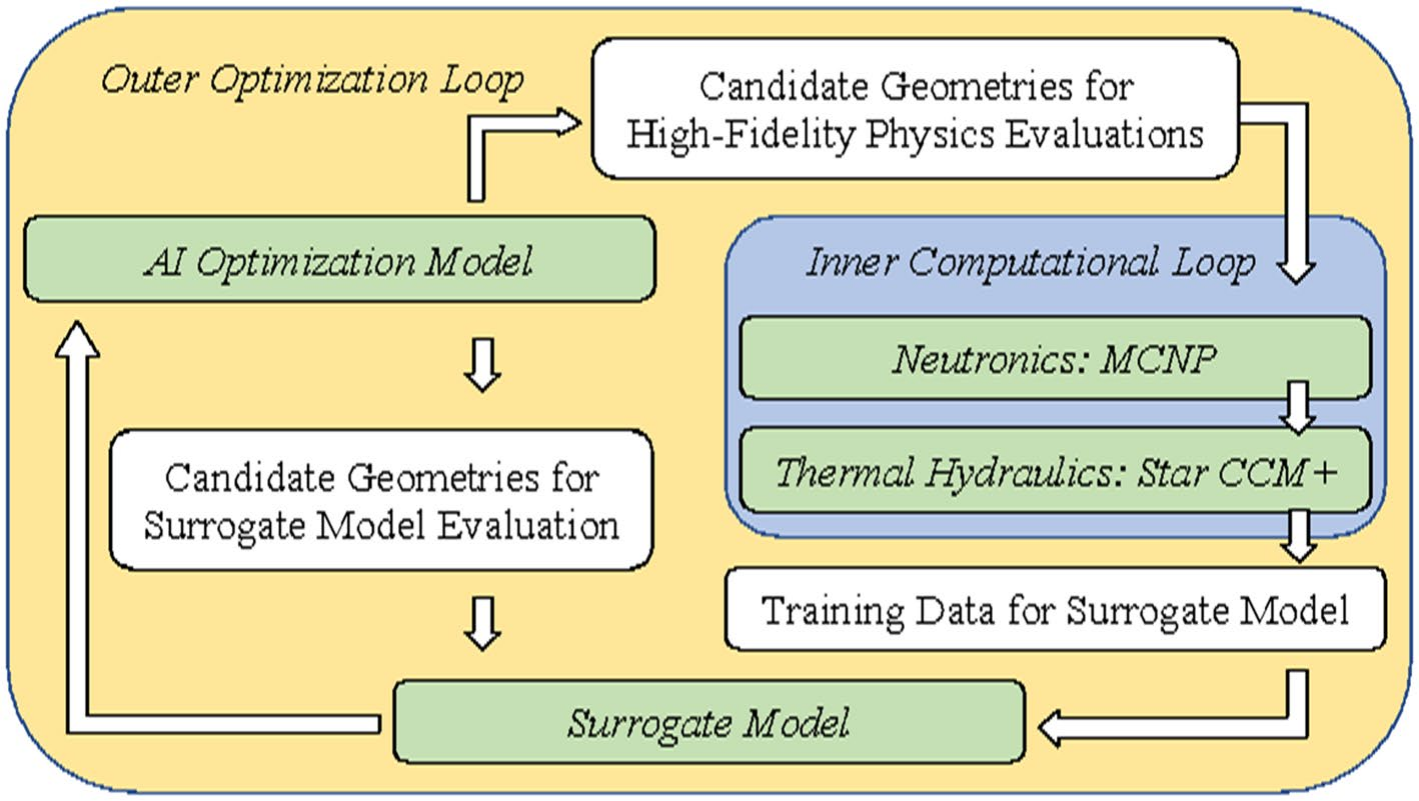
Illustration of the AI optimization workflow. Created using Microsoft PowerPoint, version 16.52, www.microsoft.com.

The authors identified two categories of tasks necessary for successfully developing a holistic AI-based approach to computationally optimizing nuclear systems. The tasks are separated into the *inner computational loop* and the *outer optimization loop.* The inner computational loop involves the full-fidelity physics simulation of the candidate designs needed to create the training data for the multiphysics ML-based emulator. The outer optimization loop begins with training the emulator based on the results generated with the full-fidelity physics calculations, and design parameters are chosen from a random sample of the design space. The outer optimization loop is the AI model for the design space that is updated based on massively parallel evaluations of the multiphysics emulator on thousands of candidate geometries. Figure 2 illustrates the workflow. The outer loop can be thought of as an adaptive sampling method, ubiquitous in computation design^17^, where each iteration focuses on a smaller design space that minimizes the given loss function.

### Inner computational loop

The neutronic modeling for this project was conducted in the Monte Carlo N-Particle code^19^. This code was chosen due to its ability to calculate power in nonfuel materials. This allowed for a very accurate power density profile in all materials. To save on computational time, the model was 30° of the full core (1/12) with reflective boundary conditions (i.e., instead of the full 360°). Because of the intrinsic symmetry and core optimization variables, this model represents a full-core model. The model was run with four tally meshes to account for the energy deposition in a cell from neutrons and photons in the fuel and moderator materials. A tally was produced for each mesh bin. The mesh bin density was 504 in the *x* direction, 252 in the *y* direction, and 20 in the *z* direction. Therefore, the lengths of each mesh bin were 0.1, 0.1, and 4 cm. These sizes were determined because they have a reasonable computational cost neutronically and thermofluidically, and meshing studies determined that they were sufficiently fine to achieve good resolution on the fuel annulus and coolant channels.

There were 750 cycles run with 10,000 particles in each cycle. The first 50 cycles were inactive which means they were not taken into account for the determination of k-effective, flux, or reaction rates. It is necessary to have inactive cycles in the beginning of a Monte Carlo simulation to converge the fission source. The average standard deviation of k-effective was 0.00024 and the maximum was 0.00030. The power density was normalized to maintain a constant core power of 3 MW. MCNP was run in “mode N P” which accounts for neutron-induced photons. This is necessary for the photon-heating tallies.

The thermofluidics model developed for this work relies on numerical methods (e.g., temporal and spatial discretization) and physical models (e.g., turbulent flow, conjugate heat transfer) to predict the temperature and flow distribution in the geometry of interest. The inherent assumptions of these methods and models must be quantified to ensure the correctness and accuracy of the results. In view of the diverse geometry configurations of the optimized designs, an accurate prediction of temperature distribution in the component is necessary. The thermofluidic model must be capable of computing a conjugate (i.e., solid fluid) heat transfer in arbitrary geometric shapes. To achieve this, a CFD approach was taken, and the commercial software STAR-CCM+ was used^20^. This method allows complex surfaces to be discretized with finite volume techniques, as well as allows the interface between the solid structure and the coolant—gas, in this case—to be properly defined. On the solid side, a thermal diffusion of heat with a volumetric heat source is computed to determine the temperature distribution. The heat generated by nuclear fission is deposited in the core element according to the volumetric power distribution supplied by the reactor physics calculation.

On the fluid side, a Reynolds averaging of the velocity vector field was employed within the finite volume formulation. Since the assumed flow is highly turbulent, a two-equation model of turbulence, realizable k-epsilon, was used. This model is better than the standard k-epsilon model for many applications, including rotational and shear flows, and it generally gives answers that are at least as accurate^21^. The near-wall velocity field is resolved with the two-layer all wye (Y) plus method^20^. All these models accurately predict the wall heat transfer, which is critical for the proper resolution of component temperature field.

To model the geometry variation necessary for running the suite of optimization codes, a geometry parameterization method was used. Within the CFD computation, the geometry is regenerated automatically every time a new combination of parameters is tried. The software allows the computational domain to be modified—both in its geometry and discretization—without user intervention. This is achieved by automating the computing process with Java drivers. The approach is fully integrable in an autonomous workflow within the entire optimization suite.

For the thermofluidic calculation, the domain was discretized with a variable resolution but with at least four elements in the radial direction across fuel. It was meshed with a polyhedral mesh with a base dimension determined by the smallest fuel element. Because the geometry varies by fuel and channel sizes, the number of elements per each case is different and usually stays below 7 million. Some specific cases with thin fuel might increase the element count to 20 million, but this is rare. The calculations were run in parallel on 16 processes, and most run time is spent on grid generation. The average clock time for a single run is around 2 h. The cases are run until the convergence of momentum and energy residuals for at least three orders of magnitude is achieved. Sensitivity on mesh and residuals convergence was performed to allow for temperature accuracy of less than 1° to be achieved. The employed modeling approach relies on component physics validation which is part of the software qualification process implemented at ORNL. The flow and heat transfer solutions, and the corresponding modeling techniques, were initially tested and verified by standard test problems. The models will be further tested against integral experiments after such are carried out as part of a comprehensive testing program.

**Figure 3:**
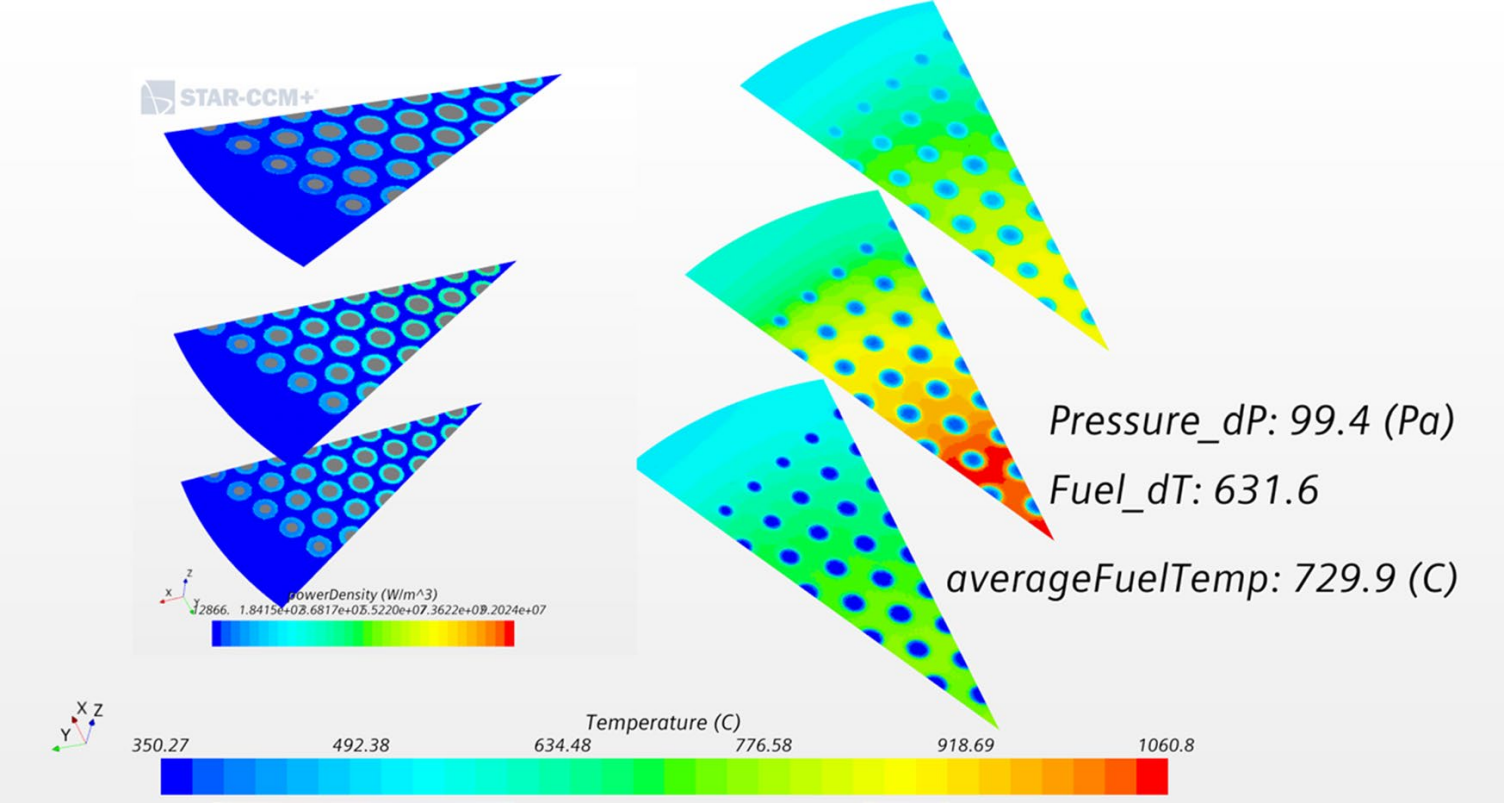
Temperature field from the thermofluidic solution. Three axial sections are plotted at lower, middle, and upper core element levels. They show the temperatures in the coolant, fuel, and moderator. On the left, the power input to the fuel and moderator is provided for reference. Both fuel and channel sizes vary in the radial direction. Created using Star-CCM+, version 2020.1, www.plm.automation.siemens.com/global/en/products/simcenter/STAR-CCM.html.

Figure 3 illustrates a typical result from the thermofluidic solution. Contours of temperatures are shown at three axial levels in the core: low (15 cm), middle (45 cm), and top (75 cm). The highest temperature is reached on the fuel-moderator boundary because the design does not involve extra moderator cooling and because the power deposited in the moderator is removed only through fuel-cooling channels. The radial variation of cooling channels is clearly visible, as defined by the optimization algorithm. Channels are larger in the central part and smaller at the periphery where the power density is lower and less coolant is needed. The average pressure drop and fuel temperature and the maximum fuel temperature difference were also calculated and are provided for reference. The input power density is plotted on the left side of Fig. 3 at the same axial positions. The simulation uses power deposition in the fuel and moderator. The power plot gives some impression for the fuel thickness variation (light blue color) with more fuel toward the periphery and less in the middle of core. Although not evident from the limited axial locations plotted, the fuel and channel sizes also vary axially as eight piece-wise linear sections (Fig. 1).

### Outer optimization loop

Simulation-based computational design can quickly become an intractable problem, depending on the size of the computational design space and computational complexity of the design simulation. The authors developed reduced-order surrogate models for neutronics and thermofluidics that can quickly sample hundreds of thousands of geometries on Summit. By using the combination of surrogate modeling and sparse validation with correction from full-physics simulation, the authors were able to train GP ML methods to accurately predict optimal designs. On average, one reactor core design takes ~ 150 s for the reduced-order surrogate model to simulate on a single Summit GPU. This time includes all setup costs and data movement. With six GPUs per node, the authors were able to test ~ 150 reactor geometries per hour per node. Generally, the surrogate is around 95% accurate compared with full physics simulations that use relative least square error measure on the objective function for this problem. Also, the surrogate model generally struggles the most at the inlet region. For the core challenge problem, Summit simulated ~ 10,000 different geometries in combination with ~ 100 full physics simulations in four iterations to determine an optimal design. We provide the code in the supplemental materials for this paper.

The outer loop AI model is based upon Gaussian processes, which are a kernel-based machine learning method that provides an efficient method for ML applicable to physics-oriented problems in engineering sciences. Specifically, given a set,

2 $$\left\{\left({p}_{i},{P}_{i}\left(x\right),{T}_{i}\left(x\right),{V}_{i,c}\left(x\right),{V}_{i,f}\left(x\right),{V}_{i,m}\left(x\right),{L}_{i},{\sigma }_{i}\right):i=1,...,N\right\}$$

of training data the loss can be determined for any parameter set. Here, *x* is the position vector, and ${p}_{i}$ is the parameter vector for the *i*th design. The functions are the power ${P}_{i}$, component temperature ${T}_{i}$, fractional coolant indicator ${V}_{i,c}$, fractional fuel indicator ${V}_{i,f}$, and fractional moderator indicator ${V}_{i,m}$ for the *N* simulated training sets. The loss ${L}_{i}$ for any design is defined to be the standard deviation of temperature for every domain with positive fuel indicator. The last item in this collection is the error estimation of the loss ${\sigma }_{i}$. The loss of any design $p$ is predicted by using the kernel-based ML method defined as:

3 $$L\left( p \right) = \sum\limits_{{i = 1}}^{N} {c_{i} k\left( {p,p_{i} } \right)}$$

where kernel function, $k\left(p,{p}_{i}\right)={e}^{-\frac{1}{2}{\| p-{p}_{i}\| }^{2}}$ is used. The coefficients of the kernel-based ML are found by solving

4 $$L_{j} = \sum\limits_{{i = 1}}^{N} {c_{i} k\left( {p_{j} ,p_{i} } \right)} \equiv Kc$$

for $j=1,...,N$ training sets, where *L*_*j*_ losses are known. The matrix elements are given as ${K}_{i,j}=k\left({p}_{i},{p}_{j}\right)$ for $1\le i,j\le N$ and the coefficient vector $c=\left({c}_{1},...,{c}_{N}\right)$.

The data from the full-fidelity physic model are augmented with a set of M emulated models:

5 $$\left\{\left({p}_{i},{P}_{i}\left(x\right),{\tilde{T }}_{i}\left(x\right),{V}_{i,c}\left(x\right),{V}_{i,f}\left(x\right),{V}_{i,m}\left(x\right),{L}_{i},{\sigma }_{i}\right):i=N+1,...,N+M\right\}.$$

The ML method assumes that the full fidelity physical models are exact, or ${\sigma }_{i}=0$ for $i=1,...,N$. In the case of $i=N+1,...,N+M$, a low-resolution approximation of the physics is used to estimate the temperature function $\tilde{T }$ by solving:

6 $$-\left({{\alpha }_{c}V}_{i,c}\left(x\right)+{{\alpha }_{f}V}_{i,f}\left(x\right)+{{\alpha }_{m}V}_{i,m}\left(x\right)\right)\Delta {\tilde{T }}_{i}\left(x\right)={\upsilon }_{i}\left(x\right)\frac{\partial {\tilde{T }}_{i}\left(x\right)}{\partial x}+{P}_{i}\left(x\right)$$

where ${\upsilon }_{i}\left(x\right)$ is a flow field, $\frac{\partial {\tilde{T }}_{i}\left(x\right)}{\partial x}$ is the temperature gradient along the flow direction, and ${\alpha }_{c}$,${\alpha }_{f}$, and ${\alpha }_{m}$ are constants. For any design $p$, the flow field is calculated based on the volumetric rate of coolant. The flow field is zero in the solid material of the reactor. The flow field and constants ${\alpha }_{c}$,${\alpha }_{f}$, and ${\alpha }_{m}$ are calculated such that ${\sum }_{i=1}^{N}{\| {T}_{i}\left(x\right)-{\tilde{T }}_{i}\left(x\right)\| }_{2}$ is minimized, where *T*_*i*_*(x)* is the training set temperatures.

When the data are augmented, the Gaussian process is calculated by:

7 $$L\left( p \right) = \sum\limits_{{i = 1}}^{{N + M}} {c_{i} k\left( {p,p_{i} } \right)}$$

where the kernel function, $k\left(p,{p}_{i}\right)={e}^{-\frac{1}{2}{\| p-{p}_{i}\| }^{2}}$ is used. The coefficients of the kernel-based ML are found by solving

8 $$L_{j} = \sum\limits_{{i = 1}}^{{N + M}} {c_{i} \left( {k\left( {p_{j} ,p_{i} } \right) + \sigma _{i}^{2} \delta _{{i,j}} } \right)} \equiv Kc$$

for $j=1,...,N+M$ and ${\sigma }_{i}=0$ for $i\le N$, where the matrix elements are given as ${K}_{i,j}=k\left({p}_{i},{p}_{j}\right)$ for $1\le i,j\le N+M$, the coefficient vector $c=\left({c}_{1},...,{c}_{N+M}\right)$, and *σ*_*i*_ is an estimate for the error in the emulation.

## Results

**Figure 4:**
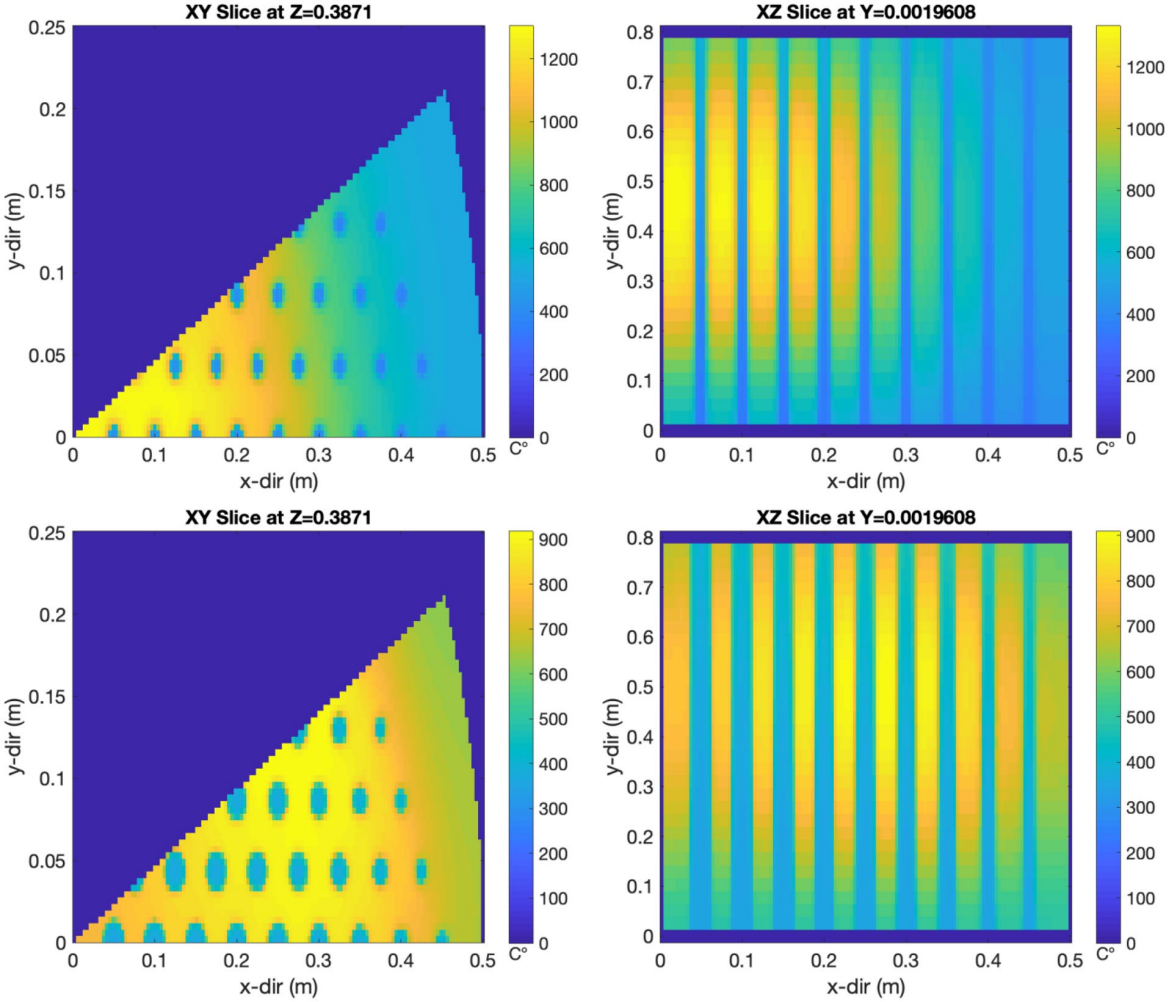
Visual representation of the temperature distribution in the original (top) and optimized (bottom) cores. The left two figures present a radial slice at an axial location of 38.7 cm out of a total core height of 80 cm. The right two figures present an axial slice through the core. Created using MATLAB R2020b, www.mathworks.com.

The quantitative objective function for the challenge problem’s optimization design was to minimize the temperature peaking in each 10 cm axial section (eight total for the full-core height) of each assembly in the core. From single-assembly simulations, the average numerical value of the objective function for a conventional design with axially uniform coolant channels of one radius was 842.1 ℃. The optimal design results in a final value of the objective function of 291.35 ℃; a 3× improvement in the objective function is achieved through the AI-based optimization of the geometry of the cooling channels when compared to the constant-cooling-channel-radius design. Figure 4 presents the visualization of the calculated temperature distribution in the core before and after the optimization.

**Figure 5:**
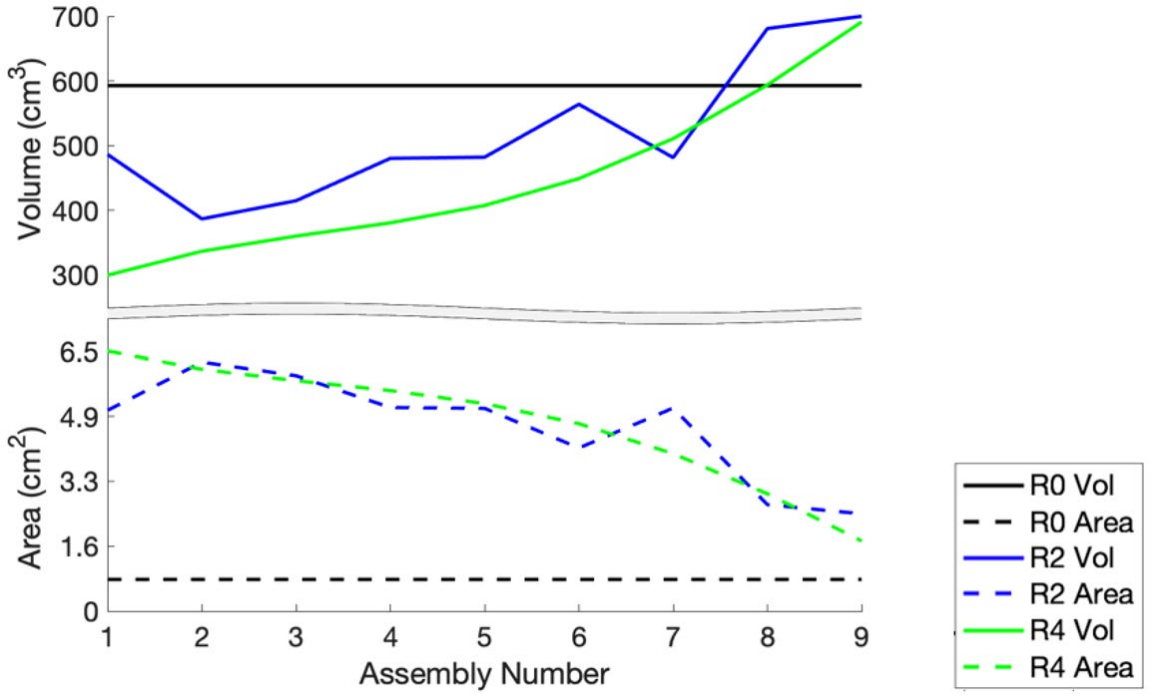
Plot of the fuel volume per assembly (top) and the heat-exchange area for the cooling channel in each assembly (bottom). The three different colors represent different iterations of the optimization algorithm: initial (R0) and two later iterations (R2 and R4). Here, R0, R2, and R4 are shorthand for iteration round zero, two, and four, respectively. Created using MATLAB R2020b, www.mathworks.com.

Figure 5 presents a physical interpretation of the optimization results by plotting the volume of the fuel in each assembly and the heat-exchange area for the cooling channels. The initial configuration with axially uniform cooling channels in all assemblies is given in black. This presents the optimal solution for a uniform cooling channel configuration. An intermediate stage of the optimization process is shown in blue, and the final, optimal design is presented in green. The intermediate result can be identified by the non-smooth behavior of the volume and surface plots across the nine radial assembly rings, whereas the converged solution displays the physically expected smooth behavior. Furthermore, there is a trade-off between the increased heat-exchange area of the cooling channel and the reduced fuel volume. Both contribute in the same direction to reducing the temperature peaking in the middle of the core that is observed with the uniform cooling channel design.

Although the optimal configuration can be justified from an engineering analysis perspective, the result is far from trivial. The right side of Fig. 1, which plots the axial cross section of the optimal design, shows that the profile of the cooling channels changes in two dimensions: axially with the height of the core and radially across the different assembly rings. The shape of the cooling channels is also unique for each of the radial assembly rings rather than being a scaled or translated version of each other. The top plot in Fig. 5 also shows a significant reduction in the fuel volume needed to maintain the reactor critical at a fixed power level compared with the traditional, uniform design. Lastly, the extension of this result is that the reactor can be operated at higher power levels for the same amount of fuel and peak fuel temperature limits.

## Discussion

### Summary of accomplishments

This paper presents the results for the AI-based design optimization of a full nuclear reactor core with arbitrary geometry cooling channels. To accelerate the optimization space search, the authors developed a ML-based multiphysics emulator capable of running efficiently on Summit. The authors demonstrated how an AI-based optimization algorithm can efficiently sample the vast and continuous search space of arbitrary geometry to find the optimal solution with significant performance improvement. In the established challenge problem, the authors demonstrated a 3× improvement in the performance metric of reducing temperature peaking factors across the core in the radial and axial directions.

### Future for nuclear design with arbitrary geometry

The authors envision a rapidly developing and promising future for nuclear systems design with arbitrary geometry. Arbitrary geometry enabled through advanced manufacturing provides a vast variety of unexplored opportunities in nuclear systems design. Historically, many engineering objectives in nuclear reactor design were met through creative combinatorial solutions of fuel loading axially and radially. Arbitrary geometry allows researchers to explore alternative solutions to these engineering challenges. Furthermore, combining variable fuel loading with elements of arbitrary geometry to maximize nuclear reactor safety and economics is an exciting opportunity.

### Future for ML-based surrogate models for rapid design evolution

The second exciting opportunity that could result from this work is the rapid design evolution that can be achieved with ML-based surrogate models, as presented in this work. The authors demonstrated that it is possible to construct ML-based surrogate models capable of capturing a large percentage of the system physics but that can be evaluated to predict the performance of candidate designs at a fraction of the computational time. This step is absolutely necessary to progress to searching larger and larger design spaces that allow for more complex nuclear system geometries.

### Future for AI-based nuclear design

The authors do not anticipate that the AI-based nuclear systems design will completely replace human designers but rather anticipate that AI-based design will become one of the main tools of the human designer. In this case, the way in which engineers think about the design problem must shift. The new focus must be to carefully craft the parameters of the optimization problem and establish the objective and constraints. The accurate formulation of the objectives will be vital, especially finding the right balance between multiple objectives. The parameters of the optimization must be chosen very carefully to reduce the possible design space as much as possible while maintaining enough flexibility to allow for significant performance improvements.

Although many challenges undoubtedly still remain, the combination of (1) arbitrary geometry enabled by advanced manufacturing, (2) ML-based surrogate models for fast and predictive computational evaluation, and (3) AI-based optimization algorithms form a very exciting future for nuclear design with the potential to discover revolutionary changes in the safety, efficiency, and economy of nuclear systems helping contribute to safer and cleaner energy for the world.

## Supplementary Information

Supplementary Information.

## References

[1] Oak Ridge National Laboratory. Transformational challenge reactor program. https://tcr.ornl.gov/ (2021). Accessed 17 August 2021.

[2] Sobes, V., *et al*. Artificial intelligence design of nuclear systems empowered by advanced manufacturing. In *PHYSOR 2020—Transition to a Scalable Nuclear Future*, Cambridge, United Kingdom, March 29–April 2, 2020.

[3] B. R. Betzler, B. J. Ade, *et al*. Advanced manufacturing for nuclear core design. In *PHYSOR 2020—Transition to a Scalable Nuclear Future*, Cambridge, United Kingdom, March 29–April 2, 2020.

[4] Oak Ridge Leadership Computing Facility, “Summit: America’s Newest and Smartest Supercomputer,”. https://www.olcf.ornl.gov/Summit/ (2021). Accessed 17 August 2021.

[5] Betzler BR, Ade BJ (2019). Advanced Manufacturing for Nuclear Core Design.

[6] Wilding PR, Murray NR, Memmott MJ (2020). The use of multi-objective optimization to improve the design process of nuclear power plant systems. Ann. Nucl. Energy.

[7] Pereira CMNA, Lapa CMF (2003). Coarse-grained parallel genetic algorithm applied to a nuclear reactor core design optimization problem. Ann. Nucl. Energy.

[8] Pereira CMNA, Schirru R, Martinez AS (1999). Basic investigations related to genetic algorithms in core designs. Ann. Nucl. Energy.

[9] Jayalal ML, Ramachandran S, Rathakrishnan S, Satya Murty SAV, Sai Baba M (2015). Application of genetic algorithm methodologies in fuel bundle burnup optimization of pressurized heavy water reactor. Nucl. Eng. Design.

[10] Pazirandeh A, Tayefi S (2012). Optimizing the fuel management in a VVER-1000 reactor using an artificial neural network. Ann. Nucl. Energy.

[11] Zameer A, Mirza SM, Mirza NM (2014). Core loading pattern optimization of a typical two-loop 300MWe PWR using simulated annealing (SA), novel crossover genetic algorithms (GA) and hybrid GA(SA) schemes. Ann. Nucl. Energy.

[12] Gomez-Fernandez M, Higley K, Tokuhiro A, Welter K, Wong WK, Yang H (2020). Status of research and development of learning-based approaches in nuclear science and engineering: A review. Nucl. Eng. Des..

[13] Liu Z, Wang J, Tan S, Qiao S, Ding H (2019). Multi-objective optimal design of the nuclear reactor pressurizer. Int. J. Adv. Nucl. Reactor Design Technol..

[14] Betzler BR, Chandler D, Cook DH, Davidson EE, Ilas G (2019). Design optimization methods for high-performance research reactor core design. Nucl. Eng. Des..

[15] J. Pevey, O. Chvala, S. Davis, V. Sobes, W.Hines Genetic algorithm design of a coupled fast and thermal subcritical assembly. *Nucl. Technol.* **206**(4) (2020).

[16] A. Archibald, V. Sobes, B. Hiscox, E. Popov, *et al*. Physics based machine learning for HPC computational design. In *Conference on Data Analysis 2020*, Santa Fe, New Mexico, February 26, 2020.

[17] Forrester A, Sobester A, Keane A (2008). Engineering Design Via Surrogate Modelling: A Practical Guide.

[18] Williams CKI, Rasmussen CE (2006). Gaussian Processes for Machine Learning.

[19] *MCNP—A General Monte Carlo N-Particle Transport Code, Version 5*, Los Alamos National Laboratory, Vol. I, 2–71 (**2–80**) (2005).

[20] Siemens. Simcenter STAR-CCM+ software. https://www.plm.automation.siemens.com/global/en/products/simcenter/STAR-CCM.html.

[21] ANSYS, Inc. *ANSYS-12.0 Theory Guide* (2009).

