# Supplementary material for: AI-based design of a nuclear reactor core
Source: Sci Rep. 2021 Oct 4;11:19646. doi: 10.1038/s41598-021-98037-1 (PMC8490470; doi:10.1038/s41598-021-98037-1)
Supplement: Supplementary file 1 — Supplementary Information. [file 41598_2021_98037_MOESM1_ESM.zip › Outer Loop Detail.docx]

The outer loop AI model is based upon Gaussian process, which are a kernel-based machine learning method that provides an efficient method for ML applicable to physics-oriented problems in engineering sciences. Specifically, given a set

$\left\{ \left( p_{i},P_{i}\left( x \right),T_{i}\left( x \right),V_{i,c}\left( x \right),V_{i,f}\left( x \right),V_{i,m}\left( x \right),L_{i},\sigma_{i} \right):i=1,...,N \right\}$ (1)

of training data the loss can be determined for any parameter set. Here, *x* is the position vector, and $p_{i}$ is the parameter vector for the *i^th^* design. The functions are the power $P_{i}$, component temperature $T_{i}$, fractional coolant indicator $V_{i,c}$, fractional fuel indicator $V_{i,f}$, and fractional moderator indicator $V_{i,m}$ for the *N* simulated training sets. The loss $L_{i}$ for any design is defined to be the standard deviation of temperature for every domain with positive fuel indicator. The last item in this collection is the error estimation of the loss $\sigma_{i}$. The loss of any design $p$ is predicted by using the kernel-based ML method defined as:

$L\left( p \right)=\sum_{i=1}^{N} c_{i}k\left( p,p_{i} \right)$ (2)

where kernel function, $k\left( p,p_{i} \right)=e^{-\frac{1}{2}\left\| p-p_{i} \right\|^{2}}$is used. The coefficients of the kernel-based ML are found by solving

$L_{j}=\sum_{i=1}^{N} c_{i}k\left( p_{j},p_{i} \right)\equiv\boldsymbol{Kc}$ (3)

for $j=1,...,N$ training sets, where *L_j_* losses are known. The matrix elements are given as $\boldsymbol{K}_{\boldsymbol{i,j}}\boldsymbol{=}k\left( p_{i},p_{j} \right)$ for $1\leq i,j\leq N$ and the coefficient vector $\boldsymbol{c}=\left( c_{1},...,c_{N} \right)$.

The data from the full-fidelity physic model are augmented with a set of M emulated models:

$\left\{ \left( p_{i},P_{i}\left( x \right),\tilde{T}_{i}\left( x \right),V_{i,c}\left( x \right),V_{i,f}\left( x \right),V_{i,m}\left( x \right),L_{i},\sigma_{i} \right):i=N+1,...,N+M \right\}$. (5)

The ML method assumes that the full fidelity physical models are exact, or $\sigma_{i}=0$ for $i=1,...,N$. In the case of $i=N+1,...,N+M$, a low-resolution approximation of the physics is used to estimate the temperature function $\tilde{T}$ by solving:

$-\left( {\alpha_{c}V}_{i,c}\left( x \right)+{\alpha_{f}V}_{i,f}\left( x \right)+{\alpha_{m}V}_{i,m}\left( x \right) \right)\Delta\tilde{T}_{i}\left( x \right)=\upsilon_{i}\left( x \right)\frac{\partial\tilde{T}_{i}\left( x \right)}{\partial x}+P_{i}\left( x \right)$ (6)

where $\upsilon_{i}\left( x \right)$ is a flow field, $\frac{\partial\tilde{T}_{i}\left( x \right)}{\partial x}$ is the temperature gradient along the flow direction, and $\alpha_{c}$,$\alpha_{f}$, and $\alpha_{m}$ are constants. For any design $p$, the flow field is calculated based on the volumetric rate of coolant. The flow field is zero in the solid material of the reactor. The flow field and constants $\alpha_{c}$,$\alpha_{f}$, and $\alpha_{m}$ are calculated such that $\sum_{i=1}^{N} \left\| T_{i}\left( x \right)-\tilde{T}_{i}\left( x \right) \right\|_{2}$is minimized, where *T_i_(x)* is the training set temperatures.

When the data are augmented, the Gaussian process is calculated by:

$L\left( p \right)=\sum_{i=1}^{N+M} c_{i}k\left( p,p_{i} \right)$ (7)

where the kernel function, $k\left( p,p_{i} \right)=e^{-\frac{1}{2}\left\| p-p_{i} \right\|^{2}}$is used. The coefficients of the kernel-based ML are found by solving

$L_{j}=\sum_{i=1}^{N+M} c_{i}\left( k\left( p_{j},p_{i} \right)+\sigma_{i}^{2}\delta_{i,j} \right)\equiv\boldsymbol{Kc}$ (8)

for $j=1,...,N+M$ and $\sigma_{i}=0$ for $i\leq N$, where the matrix elements are given as $\boldsymbol{K}_{\boldsymbol{i,j}}\boldsymbol{=}k\left( p_{i},p_{j} \right)$ for $1\leq i,j\leq N+M$, the coefficient vector $\boldsymbol{c}=\left( c_{1},...,c_{N+M} \right)$, and *σ_i_* is an estimate for the error in the emulation.
